# Don’t forget about tau: the effects of ApoE4 genotype on Alzheimer’s disease cerebrospinal fluid biomarkers in subjects with mild cognitive impairment—data from the Dementia Competence Network

**DOI:** 10.1007/s00702-022-02461-0

**Published:** 2022-01-21

**Authors:** Gloria S. Benson, Chris Bauer, Lucrezia Hausner, Samuel Couturier, Piotr Lewczuk, Oliver Peters, Michael Hüll, Holger Jahn, Frank Jessen, Johannes Pantel, Stefan J. Teipel, Michael Wagner, Johannes Schuchhardt, Jens Wiltfang, Johannes Kornhuber, Lutz Frölich

**Affiliations:** 1 Department of Geriatric Psychiatry, Medical Faculty Mannheim, Central Institute of Mental Health, Heidelberg University, Mannheim, Germany; 2 MicroDiscovery GmbH, Berlin, Germany; 3 Department of Psychiatry and Psychotherapy, Friedrich-Alexander-University of Erlangen-Nuremberg, Nuremberg, Germany; 4 Department of Neurodegeneration Diagnostics, Medical University of Bialystok, Bialystok, Poland; 5 Department of Psychiatry and Psychotherapy, Campus Benjamin Franklin, Charité Berlin, Berlin, Germany; 6 Clinic for Geriatric Psychiatry and Psychotherapy Emmendingen and Department of Psychiatry and Psychotherapy, Center for Psychiatry, University of Freiburg, Freiburg, Germany; 7 Department of Psychiatry and Psychotherapy, University Medical Center Hamburg, Hamburg, Germany; 8 Department of Psychiatry and Psychotherapy, Medical Faculty, University of Cologne, Cologne, Germany; 9 Department of Psychiatry and Psychotherapy, University of Bonn, Bonn, Germany; 10 German Center for Neurodegenerative Diseases (DZNE), Cologne/Bonn, Bonn, Germany; 11 Ageing Medicine, Institute of General Practice, Goethe University, Frankfurt, Germany; 12 Department of Psychiatry and Psychotherapy, Ludwig-Maximilian-University of Munich, Munich, Germany; 13 Department of Psychosomatic Medicine, University Medicine Rostock, Rostock, Germany; 14 German Center for Neurodegenerative Diseases (DZNE), Rostock, Germany; 15 Department of Psychiatry and Psychotherapy, University Medical Center Göttingen, Göttingen, Germany; 16 German Center for Neurodegenerative Diseases (DZNE), Göttingen, Germany

**Keywords:** Apolipoprotein E, Alzheimer’s disease, Mild cognitive impairment, Amyloid beta42, Total tau protein, Phopho-tau protein, CSF biomarkers

## Abstract

﻿ApoE4, the strongest genetic risk factor for Alzheimer’s disease (AD), has been shown to be associated with both beta-amyloid (Aβ) and tau pathology, with the strongest evidence for effects on Aβ, while the association between ApoE4 and tau pathology remains inconsistent. This study aimed to investigate the associations between ApoE4 with CSF Aβ42, total tau (t-tau), phospho-tau181 (p-tau), and with the progression of decline in a large cohort of MCI subjects, both progressors to AD and other dementias, as well as non-progressors. We analyzed associations of CSF Aβ42, p-tau and t-tau with ApoE4 allele frequency cross-sectionally and longitudinally over 3 years of follow-up in 195 individuals with a diagnosis of MCI-stable, MCI-AD converters and MCI progressing to other dementias from the German Dementia Competence Network. In the total sample, ApoE4 carriers had lower concentrations of CSF Aβ42, and increased concentrations of t-tau and p-tau compared to non-carriers in a gene dose-dependent manner. Comparisons of these associations stratified by MCI-progression groups showed a significant influence of ApoE4 carriership and diagnostic group on all CSF biomarker levels. The effect of ApoE4 was present in MCI-stable individuals but not in the other groups, with ApoE4 + carriers having decreased CSF Aβ 42 levels, and increased concentration of t-tau and p-tau. Longitudinally, individuals with abnormal t-tau and Aβ42 had a more rapid progression of cognitive and clinical decline, independently of ApoE4 genotype. Overall, our results contribute to an emerging framework in which ApoE4 involves mechanisms associated with both CSF amyloid-β burden and tau aggregation at specific time points in AD pathogenesis.

## Background

Alzheimer’s disease (AD), the most frequent neurodegenerative disease, is characterized by an accumulation of extracellular beta-amyloid (Aβ) plaques and intracellular tau tangles in the brain. Its pathobiology is multifactorial with both genetic and environmental risk factors (Scheltens et al. 2021). According to epidemiological and genome-wide association studies, apolipoprotein E4 (ApoE4) is the greatest single genetic risk factor for late-onset AD sporadic (Corder et al. 1993; de Rojas et al. 2021). Three common polymorphisms in the ApoE gene, ɛ2, ɛ3, and ɛ4, result in a single amino acid change in the ApoE protein. ApoE ɛ2, ɛ3, and ɛ4 alleles strongly alter, in a dose-dependent manner, the likelihood of manifesting Alzheimer's disease and cerebral amyloid angiopathy (Verghese et al. 2011). Heterozygous ApoE4 carriers have an approximately fourfold increase of risk compared with the most prevalent homozygous carriers of the ε3 allele, whereas in homozygous ApoE4 carriers, the increase of risk is approximately 12-fold (Holtzman et al. 2012).

Cerebrospinal fluid (CSF) biomarkers, such as different species of amyloid-β (Aβ), total tau (t-tau) and phosphorylated tau (p-tau), have been proven to be of great diagnostic value in the early diagnosis of AD (Lewczuk et al. 2018). The accumulation of the Aβ42 peptide (Aβ42) and its aggregated forms is hypothesized to be the initial trigger of Alzheimer pathology and may be used as a diagnostic and prognostic biomarker (Selkoe and Hardy 2016; Hansson 2021). Decreased concentrations of CSF Aβ42 are indicative for cerebral amyloid pathology across the entire continuum of AD, from preclinical asymptomatic stage to dementia stage (Vos et al. 2015). An association between the ApoE4 genotype and CSF concentrations of Aβ42 has been described for AD patients and healthy controls, with the ApoE4 allele being associated with lower CSF Aβ42 concentrations in a gene dose-dependent manner (Galasko et al. 1998; Vemuri et al. 2009; Lautner et al. 2014; Konijnenberg et al. 2020).

Measurement of tau protein in the CSF is also used as a biomarker in AD and is considered to be linked to neurodegeneration (van Rossum et al. 2012; Frölich et al. 2017). In particular, hyperphosphorylated isoforms of tau, e.g., tau protein phosphorylated at threonine181 is the gold standard for tau CSF biomarkers that are used to diagnose AD (Janelidze et al. 2020). A recent study using quantitative mass spectrometry demonstrated that phosphorylation at threonine 217 may be a more sensitive marker (Karikari et al. 2021). While the associations between ApoE4 and CSF Aβ42 have been robustly reported, the associations between ApoE4 in CSF t-tau and p-tau remain inconsistent (Galasko et al. 1998; Herukka et al. 2007; Vemuri et al. 2009; Morris et al. 2010; Risacher et al. 2013). It remains a matter of debate, if the associations between the ApoE4 genotype and CSF concentrations of total tau and or phosphorylated tau protein concentrations and the progression of cognitive decline are of similar magnitude and validity as the association between the ApoE4 genotype and CSF concentrations of Aβ42 in AD. More specifically, it is unclear if the ApoE4 effects may still be evident at a stage of AD, when mechanisms of neurodegeneration are most pronounced, e.g., at the stage of mild cognitive impairment (MCI).

The aim of the present study was to analyze the ApoE4 allele frequency in interaction with CSF concentrations of Aβ42, t-tau and p-tau in a large heterogeneous sample of MCI patients followed longitudinally with different progression outcomes: MCI-stable, MCI-AD converters and MCI progressing to other forms of dementias.

## Methods

### Subjects

The dataset we analyzed is from a prospective multisite longitudinal observational study on memory clinic patients with MCI or early dementia obtained from the Dementia Competence Network (DCN), (Kornhuber et al. 2009). The procedures for recruitment diagnosis, assessments have been published elsewhere (Kornhuber et al. 2009; Frölich et al. 2017). Individuals were selected from the cohort based on the availability of baseline CSF sample, ApoE4 genotype, at least 12 months of follow-up, outcome MCI-stable, progression to AD-only, or progression to other dementias and cognition data. Diagnoses were based on the clinical classification at follow-up and were either classified as MCI-stable (mean follow-up 25.7 months), MCI-AD or MCI-other. All individuals were clinically evaluated every 12 months up to 36 months.

The study was approved by the ethics review board of the coordinating center and by the local ethics committees and was conducted in accordance with the Declaration of Helsinki. All sub- jects gave written informed consent.

**Fig. 1:**
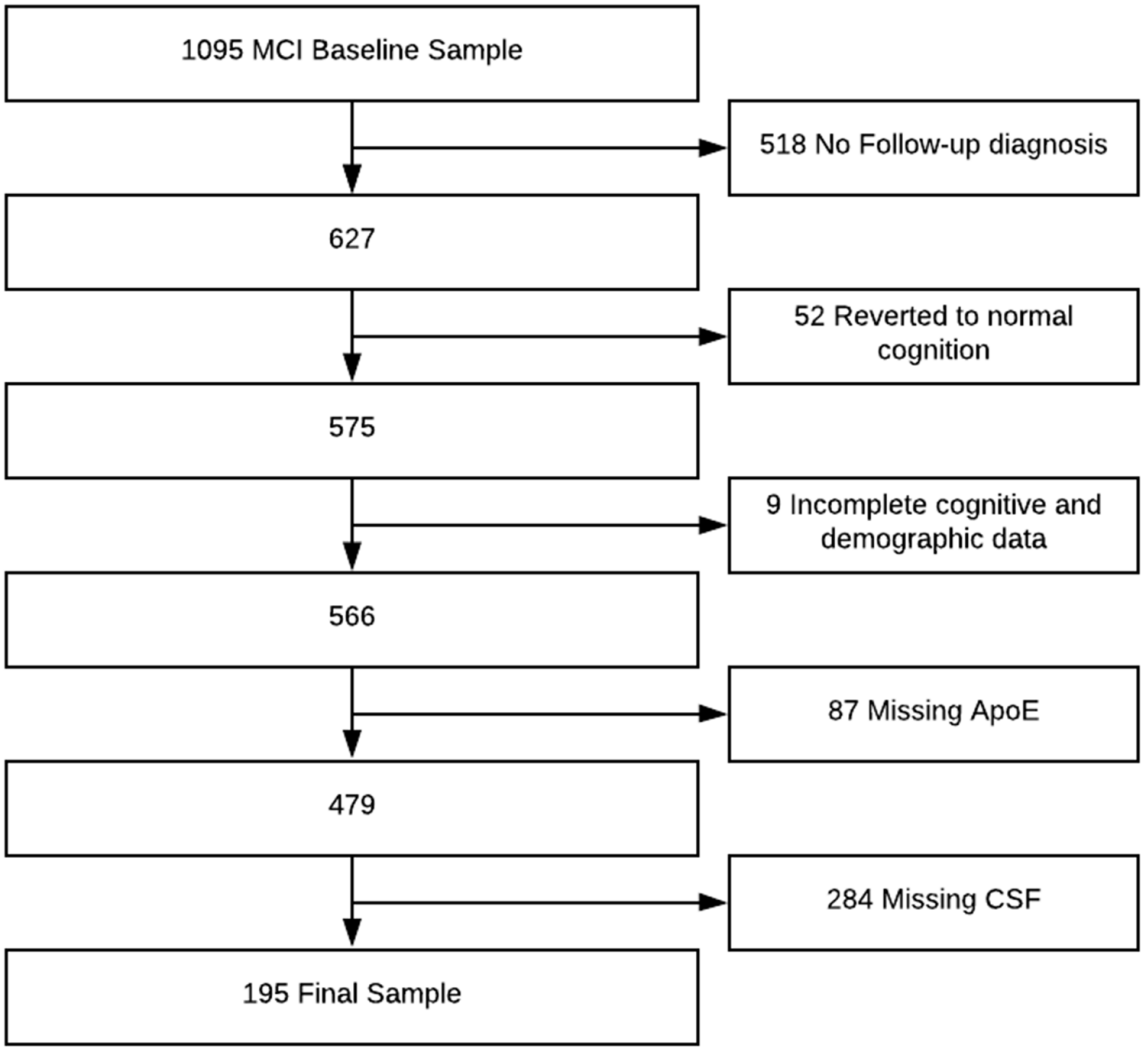
Study flow chart

For the present study, we selected those participants from the total sample of 1095 subjects with MCI at baseline in whom all relevant variables were available (see Fig. 1).

### Clinical diagnosis and assessment

The clinical assessment is described in detail in a previous publication (Kornhuber et al. 2009). In short, MCI was defined as having complaints of ﻿a cognitive deficit and objectified decline of cognitive abilities (more than 1 SD below age- and education-adjusted norms) in at least one neuropsychological domain of CERAD (Consortium to Establish a Registry of Dementia (CERAD) neuropsychological test battery) (Morris et al. 1989); no or only minor changes in complex activities of daily living (ADL) (Hindmarch et al. 1998), no major depressive episode (Montgomery and Asberg 1979). MCI patients were included at baseline and were evaluated clinically every 12 months to 36 months or until progression to incident dementia. According to DCN protocol, MCI groups were divided into outcomes subgroups, MCI-stable, progression to AD-only (MCI-AD), and progression to other dementias (MCI-Other). Stable MCI patients were defined as those with stable MCI diagnosis and no dementia at each follow-up (Clinical Dementia Rating (CDR) < 1), a Mini-Mental State Examination (MMSE) score > 24 at last follow-up visit, and a B-ADL score < 4 at each follow-up. Progressions to AD were defined as newly occurring impairments in instrumental or basic activities of daily living, as assessed by clinical protocols and international guidelines. MCI patients who developed non-AD dementia at follow-up were diagnosed using local routine clinical protocols from each site with biomarker and clinical procedures, these other dementias include (Lewy-Body, Fronto-temporal lobe dementia (FTLD) mixed Dementia and Vascular Dementia).

For the purpose of this study, we used the MMSE (Folstein et al. 1975) as a main cognitive outcome measure, and the Clinical Dementia Rating Sum of boxes (CDR-sb) as a proxy of clinical progression (Morris 1993).

### Analyses of CSF biomarkers and genetic analysis

The following CSF biomarkers were measured by enzyme-linked immunosorbent assay (ELISA): amyloid-beta1-40 (Aβ40; The Genetics Co., Zürich, Switzerland), Aβ42, total tau (t-tau), and phosphorylated tau181 (p-tau; Innogenetics, Ghent, Belgium). The analyses were performed in a certified laboratory and under a routine quality control regime (intra-assay coefficients of variation: 2.3–5.9%; inter-assay coefficients of variation: 9.8–13.7%) (Lewczuk et al. 2006). The technicians were blinded to the clinical diagnoses and other clinical information.

The ApoE4 genotyping was performed using leukocyte DNA obtained from blood samples using the Qiagen blood isolation kit (Qiagen, Hilden, Germany). The apolipoprotein ε4 genotype was determined as previously described (Hixson and Vernier 1990). Results were dichotomized into ApoE4 allele carrier (ApoE4+) or noncarrier (ApoE4−) status.

### Statistical analysis

Biomarkers were tested for normal distribution using Shapiro–Wilk normality test. Since CSF biomarkers were found to be log-normally distributed, corresponding plots are shown on logarithmic axes and corresponding *p *values are calculated assuming a log-normal distribution. For the calculation of baseline statistics for the three groups, we used the *f*-test if the variable was numerical or Kruskal–Wallis rank-sum test if the variable was categorical.

For pairwise comparison of two groups, we used the two-sample t tests with Welch's modification. For assessing effects of ApoE4 and the diagnostic group on the CSF biomarkers, we performed an ANOVA (Analysis of Variance). Besides the single variables, we also assessed the interaction effect (CSF ~ ApoE4 * group). The ANOVA model was also stratified for age and gender. To assess the effects of ApoE4 and the level of CSF markers on cognitive decline, the CSF markers were dichotomized based on the median value: 356 for total-τ, 52 for phospho-τ and 681 for Aβ-42. Cognitive decline was quantified by calculating the slopes of a linear model of CDR or MMSE over time for each patient individually. ANOVA analysis was performed to assess the effects of ApoE4 and CSF markers on cognitive decline (Cog. Decline ~ ApoE4 * CSF group). The ANOVA model was also stratified for age and gender. All statistical analyses were performed with R version 3.5.1 (R Core Team 2018).

## Results

### Demographics and biomarkers values

We assessed 195 individuals who were on average 65.28 (8.74) years old and had an average of 9.57 (1.91) years of education, with 61.03% of them being female. Clinical follow-up data were available with an average follow-up length of 25.7 month. Table 1 shows the baseline characteristics and biomarker values per group. Of the 195 MCI individuals, 49 progressed to AD Dementia (25.12%) (MCI-AD); 127 remained MCI (65.12%); and 19 individuals progressed to other forms of dementia (9.74%) (see Table 1 for group differences). The mean follow-up time was similar within all groups (25.96 month for MCI-AD, 25.42 for MCI-stable and 27.16 for MCI-other; Kruskal–Wallis PV: 0.8127). There were no significant differences in gender distribution and years of education. Age, MMSE score and CDR-sum of boxes differed significantly among the groups.

**Table 1:** Baseline characteristics and CSF biomarker values across the diagnostic groups

Variables	Overall	MCI-Stable	MCI-AD	MCI-Other	*P* values
	*N* = 195	*N* = 127	*N* = 49	*N* = 19	
Age	65.28 (8.74)	65.58 (9)	68.18 (8.05)	62.42(7.07)	0.015
Education	9.57 (1.91)	9.71 (1.94)	9.24 (1.74)	9.47 (2.12)	0.35
Sex	61.03%	62%	53%	68.42%	0.38
FU time	25.7 month	25.42 month	25.96 month	27.16 month	0.81
ApoE4 Carrier, *n* ε4(+ /–; + / +),	79 (66/13)	48 (43/5)	24 (16/2)	7 (7/0)	0.37
Aβ-42*	686.27 (302.82)	765.99 (310.62)	512.46 (185.02)	688.47 (528.86)	< 0.001
p-tau*	56.18 (28.49)	50.39 (22.68)	77.67 (34.23)	50.44 (49.08)	< 0.001
t-tau*	356.87 (217.06)	302.94 (165.6)	558.65 (258.45)	335.83 (370.49)	< 0.001
MMSE	26.9 (2.4)	27.32 (2.33)	26.08 (2.29)	26.21 (2.07)	< 0.001
CDR-sb	1.95 (1.05)	1.69(0.99)	2.29 (1.08)	2.82 (0.71)	< 0.001

Data presented as mean and standard deviation, unless presented otherwise

*Aβ42* amyloid beta1-42, *AD* Alzheimer’s disease, *ApoE4* apolipoprotein E4, *CSF* cerebrospinal fluid, *p-tau* phospho-tau-181, *t-tau* total tau, *MMSE* Mini-Mental State Examination, *CDR-sb* Clinical Dementia Rating-sum of boxes

There were no significant differences regarding ApoE4 carriership status. In the total sample, sixty-six (33.8%) had one ApoE4 allele (ε4**(**+ /–), and thirteen (6.6%) were homozygous ApoE4 carriers (ε4 + / +). The baseline levels of CSF Aβ-42, t-tau and p-tau differed significantly between the groups (Table 1). The MCI-AD group, had lower CSF Aβ-42, and higher t-tau and p-tau than the other groups.

### CSF concentrations of pTAU and tTAU in relation to and APOE 4 genotype and the impact of AΒ-42 on CSF TAU

**Fig. 2:**
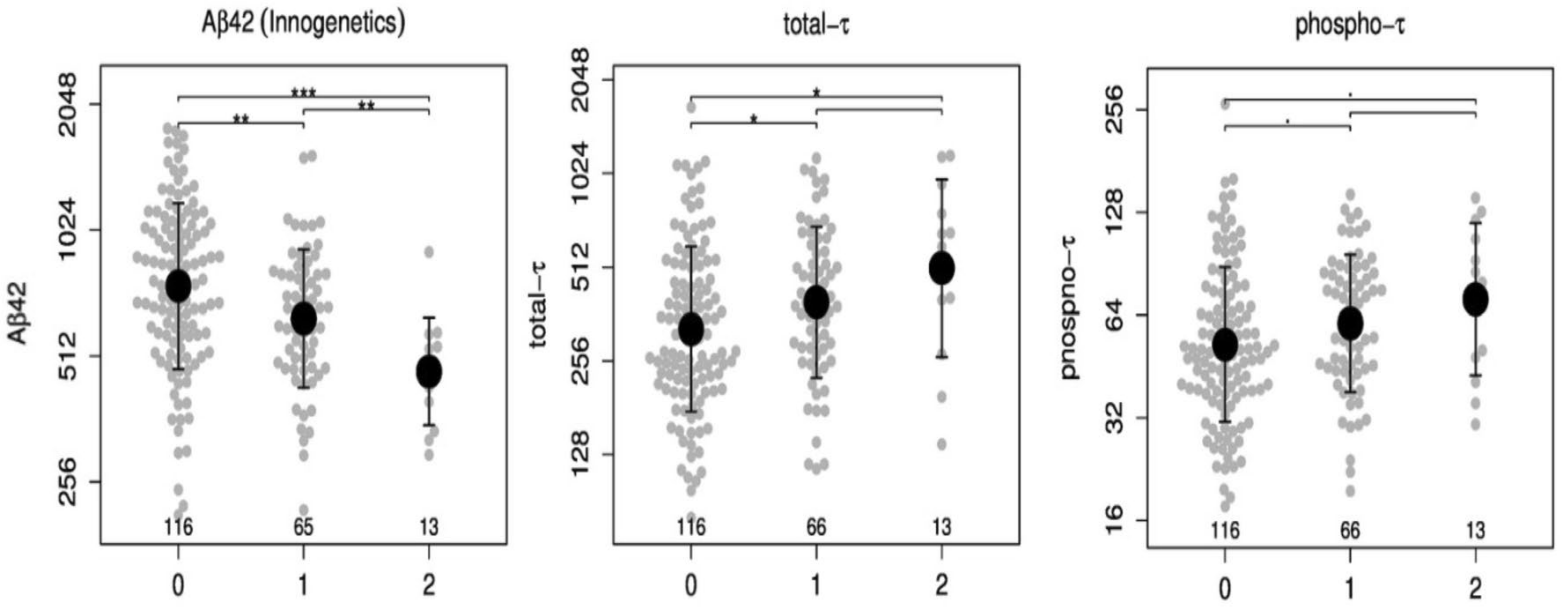
Plots (beeswarm + mean ± SD) of the three CSF AD biomarkers depending on the ApoE4 allele frequency: Aβ-42 t-tau and p-tau. CSF values are shown on logarithmic scale. *p* values were calculated and are shown above the boxes as stars (’***’*p* value < 0.001, ’**’*p*-value < 0.01, ’*’*p* value < 0.05, ’.’*p* value < 0.1). Sample sizes are given in the lower part of the plot

In the total cohort, CSF Aβ-42 concentrations were lower in ApoE4 carriers than in non-carriers in a gene dose-dependent manner (*p* < 0.001). Likewise, t-tau and p-tau were increased in ApoE4 carriers than in non-carriers in a gene dose-dependent manner (*p* < 0.01). Comparisons are shown in Fig. 2.

To further analyze the impact of amyloid pathology on CSF tau levels, we assessed the association of APOE4 genotype with CSF tau concentrations, separate for amyloid-negative and amyloid-positive individuals. To this end, we used an Aβ42 cut-off level of 500 pg/ml and performed a linear regression analysis assessing the association of APOE4 with CSF tau concentrations. The model also included the effects of the different MCI groups (MCI-stable, MCI-AD, MCI-other).

In amyloid-negative individuals (Aβ42 ≥ 500), we found a significant association between APOE epsilon4 and CSF tau (p-value: 0.02349, *N* = 147). In amyloid-positive subjects (Aβ42 < 500), there was no significant relation between APOE epsilon4 and CSF tau visible (*p*-value: 0.7, *N* = 48).

### CSF biomarkers and APOE 4 carriership stratified by MCI-progression

**Fig. 3:**
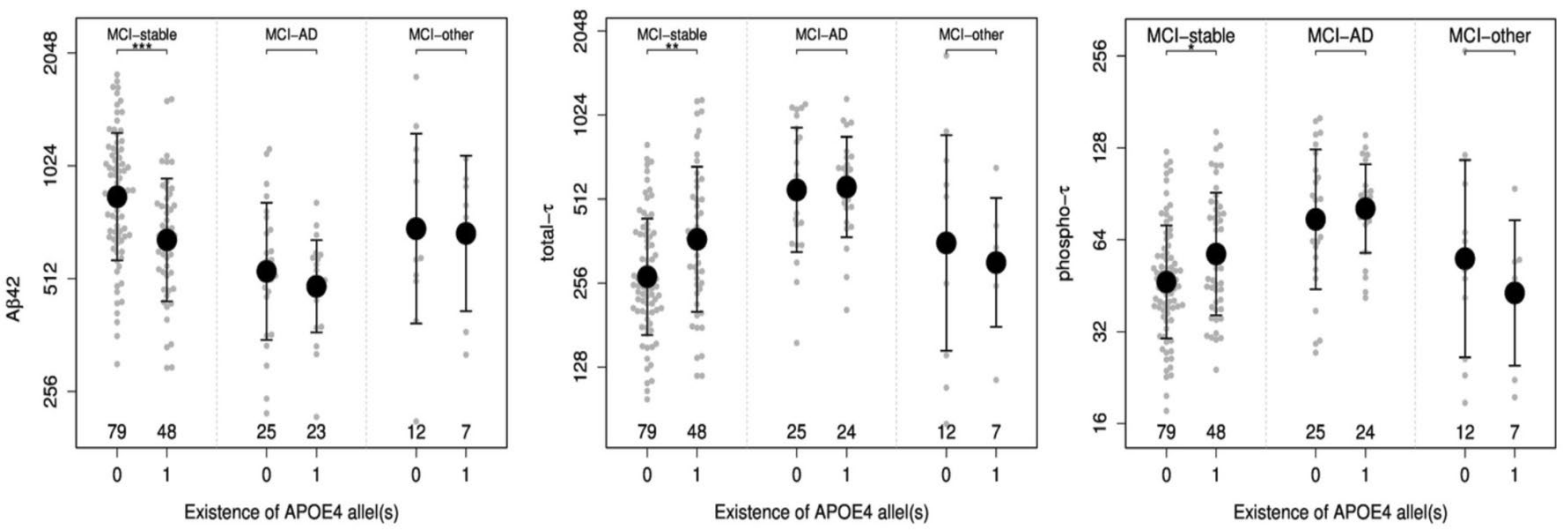
Plots (beeswarm + mean/SD) of the three CSF AD biomarkers depending on the existence of at least one ApoE4 allele: Aβ-42 t-tau and p-tau. Biomarker levels are shown on logarithmic scale. Sample sizes are given in the lower part of the plot

Comparisons by ApoE4 carriership status and MCI groups’ diagnoses of CSF concentrations are shown in Table 1. Figure 3 shows the comparisons by ApoE4 status within the MCI groups. In MCI-stable individuals, ApoE4 carriership was associated with lower levels of Aβ42 (*p* < 0.001) and increased levels of t-tau (*p* < 0.01) and p-tau (*p* < 0.05). In MCI-AD progressors and MCI-Other, no differences regarding ApoE4 carriership were found. ANOVA Models (adjusted for age and gender) show a significant influence of ApoE4 carriership and diagnosis on Aβ42, t-tau and p-tau concentrations (*p* < 0.001, *p* < 0.001, < 0.001). In addition, we also analyzed a potential combinatorial effect of ApoE4 and diagnosis. However, we found that the influence of ApoE4 was similar across all diagnosis (interaction *p * values: Aβ42 *p* = 0.45; t-tau *p* = 0.29; and p-tau *p* = 0.27).

### Progression data

**Fig. 4:**
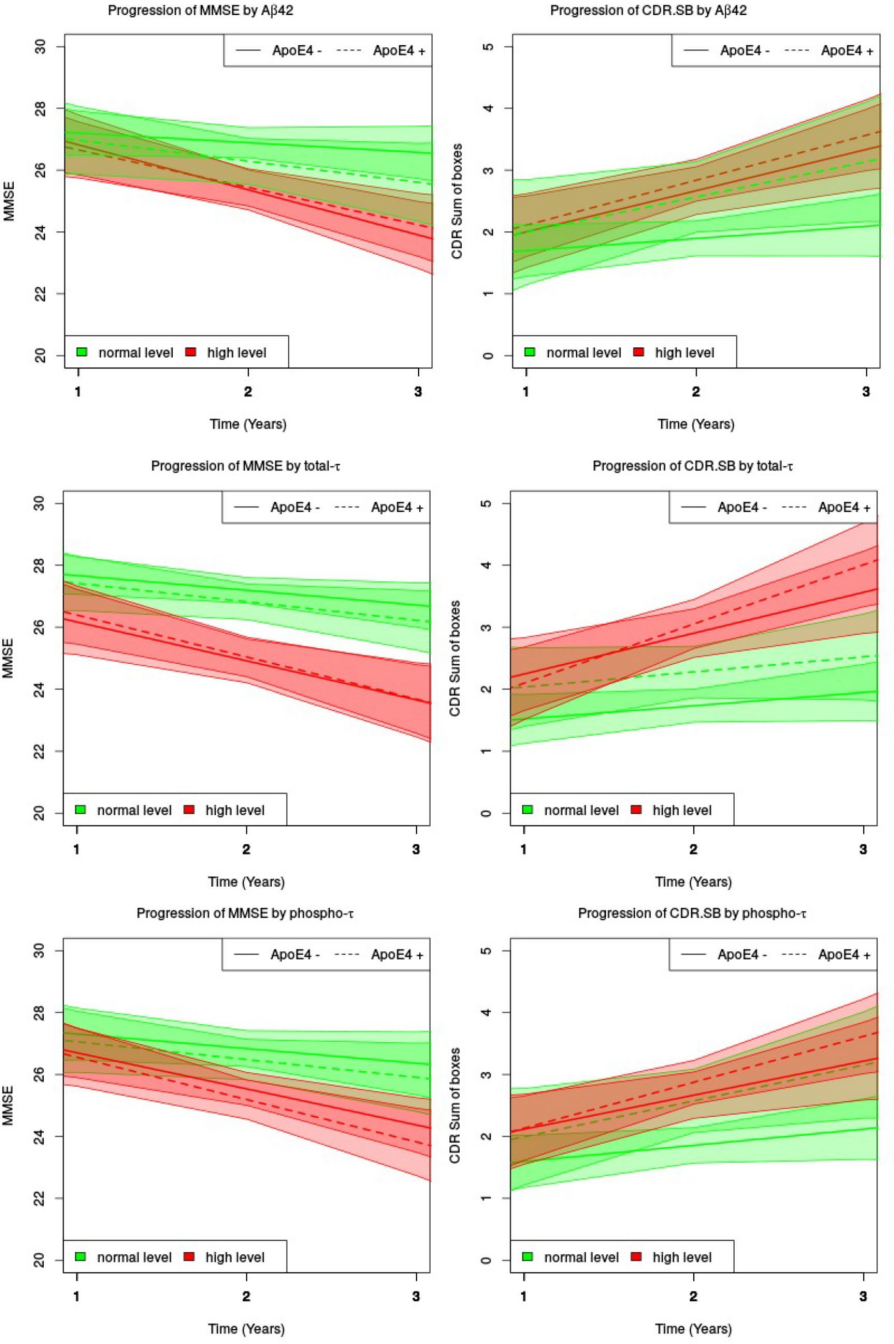
Influence of CSF Aβ-42 t-tau and p-tau on progression of cognitive performance and clinical progression stratified by ApoE4 status. The graph shows mean scores of MMSE (left) and CDR.SB (right) progression over time for high (red) and normal (green) CSF biomarker levels and by ApoE4 status (dashed lines: ApoE4 + ; solid lines: ApoE4−)

We assessed the influence of CSF markers and ApoE4 status on cognitive decline and clinical decline measured by MMSE and CDR-sb (Table 2). The analysis is stratified by ApoE4 status, and the baselines levels of CSF Aβ42, t-tau and p-tau. We found that individuals with abnormal baselines levels of t-tau and Aβ42 were significantly associated with an increased rate of cognitive decline in the total sample for MMSE (t-tau *p* ≤ 0.001; Aβ42 *p* ≤ 0.01) and for clinical progression CDR-sb (t-tau, *p* ≤ 0.001; Aβ42, 0.06) while a non-significant trend was seen for p-tau for MMSE (*p* = 0.09). ApoE4 status did not have a significant effect on rate of cognitive decline. We found no significant interaction of CSF * ApoE4 influence on the progression rate of cognitive decline. In ApoE4+ individuals, normal levels of Aβ42 seem to decline clinically similarly (CDR-sb) to those individuals with Aβ42 abnormal levels; however, this interaction did not reach significance (Interaction Aβ42* ApoE4 *p* = 0.42) (see Fig. 4).

**Table 2:** Influence of biomarkers and ApoE status and their interaction on cognitive decline and clinical progression longitudinally

	Aβ 42	t -tau	p-tau	Aβ 42	t-tau	p-tau
	MMSE	MMSE	MMSE	CDR	CDR	CDR
CSF parameter	< 0.001	< 0.001	< 0.097	0.068	< 0.001	0.219
ApoE4	﻿0.859	﻿0.886	﻿0.856	0.883	﻿0.955	﻿0.955
CSF^a^ ApoE4	﻿0.489	﻿0.623	0.942	0.428	﻿0.859	﻿0.784

ANOVA *p*-values of the dichotomized CSF parameters, ApoE4 carriership status and the combined effect

^a^Models include age and gender

## Discussion

In this study, we analyzed in detail the impact of ApoE4 allele frequency on CSF concentrations of AD core biomarkers (Aβ 42, t-tau and p-tau) cross-sectionally and on progression of decline in a cohort of MCI subjects from the Dementia Competence Network, stratified into 3 groups: MCI-stable individuals, MCI-AD progressors and MCI subjects progressing to other forms of dementia. Our results confirm a clear impact of ApoE4 on all CSF AD core biomarkers: (1) In our total sample, ApoE4 carriers had lower concentration of CSF Aβ42, and increased concentration of t-tau and p-tau than non-carriers in a gene-dose-dependent manner. (2) Comparisons of the associations stratified by MCI-progression groups show a quantitatively differential influence of ApoE4 carriership on CSF biomarkers dependent on diagnostic group. (3) In the largest group from our sample, i.e., MCI-stable individuals, CSF biomarker levels were close to normal, in line with clinical outcome, and most strongly affected by ApoE4 carrier status, i.e., decreased CSF Aβ42 levels and increased concentration of t-tau and p-tau. (4) When assessing the influence of ApoE4 and CSF biomarkers on cognitive and clinical decline over time, those individuals with abnormal t-tau and Aβ42 had a more rapid cognitive and clinical decline.

Our results add to the body of findings, showing that ApoE4 exerts a pathological influence on both Aβ42 and tau levels. However, the data also demonstrated that in clinical AD at the stage of MCI, the impact of ApoE4 is diminished by neurodegeneration. Our findings are in line with previous studies, showing an effect of ApoE4 on CSF Aβ42 and CSF tau in cognitively unimpaired subjects and early MCI, but not in AD dementia, supporting the idea that other mechanisms of neurodegeneration may override the effect of ApoE4 later in the course of AD (Herukka et al. 2007; Risacher et al. 2013; Mofrad et al. 2020). We consider this possible evidence for the apparent genetic effect on CSF biomarkers, which can become outweighed as the disease progresses. Consistent with the paradigm, in which the influence of ApoE4 leads to changes in CSF in the initial stages (before and during the phase in which patients are developing brain Aβ pathology), subsequently as frank neurodegeneration begins, there is no longer a significant increase in CSF ApoE levels as a function of increasing ApoE4 count (Berger et al. 2021). Diagnostically, this implies that the information on ApoE4 carriership in AD is already “contained” in the pathological levels of Aβ42, t-Tau and p-Tau and thus, is not diagnostically relevant at the stage of MCI (Frölich et al. 2017).

Higher levels of CSF t-tau and Aβ42 were associated with a lower cognitive performance over time and more rapid progression of decline (CDR-sb), regardless of ApoE4 status. In line with previous studies, showed the association between CSF t-tau levels and lower cognitive performance and increase rate of decline as well (Vemuri et al. 2009; Bos et al. 2019). These findings support, together with pathophysiological studies, the negative impact of t-tau on cognition. T-tau are markers of axonal degeneration; these findings imply that axonal loss may be an important driver of cognitive decline (Koutsodendris et al. 2021). We found no significant interaction of CSF Aβ42 * ApoE4 influence on the progression rate of cognitive decline, suggesting that pathological levels of these markers reflect a generic consequence of neurodegeneration regardless of ApoE4 genotype.

As expected, we confirmed the powerful ApoE4 dosage-dependent effect on CSF Aβ 42 levels reported previously (Galasko et al. 1998; Vemuri et al. 2009; Lautner et al. 2014), showing a significant negative association between ApoE4 allele number and decreased levels of Aβ42. In addition, we found a positive association between ApoE4 allele number and both CSF t-tau and p-tau levels, a finding that has been inconsistently reported in previous studies (Galasko et al. 1998; Herukka et al. 2007; Vemuri et al. 2009; Morris et al. 2010; Risacher et al. 2013).

Our analyses on the interaction between Aβ42 with APOE4 genotype on CSF tau levels may suggest that molecular processes associated with amyloid pathology “override” the effects of APOE4 on CSF tau levels in amyloid-positive subjects; thus, a significant independent effect of APOE4 genotype on CSF tau can only be demonstrated in amyloid-negative subjects.

Although the mechanisms by which ApoE4 exerts its effects on AD pathologies have been more clearly defined for Aβ (i.e., ApoE genotype affects Aβ clearance rate by slowing clearance), our results add to the growing body of recent findings, suggesting the involvement of ApoE4 on tau accumulation as well. In the case of tau pathology, ApoE4 is associated with higher levels of CSF tau (Toledo et al. 2015) and more neurofibrillary tangles at autopsy (Farfel et al. 2016), although these associations are usually relegated to individuals with high levels of amyloid pathology. However, recent longitudinal data show that tau accumulation may be accelerated in the presence of ApoE4 independent of Aβ burden (Baek et al. 2020). Additionally, an important recent study has shown an interactive effect between ApoE4 and Aβ to increase tau accumulation, as measured by Tau PET uptake (Therriault et al. 2020). Together, these findings support the interactive role of ApoE4 with both Aβ and tau in AD pathogenesis. See Koutsodendris et al. (2021) for a recent review proposing a “new multi-route pathogenic cascade for AD” whereby ApoE4 affects tau by increasing its phosphorylation and accelerating its spread to other neurons.

Our study has several limitations: (1) Our findings may be affected by a sample size bias, as we chose to include only participants with complete data in the longitudinal analysis, the sample used was rather small for some of the diagnostic groups. This may also reflect that ApoE4 effects were most pronounced in the largest group, and least clear in the smallest group. Alternatively, the findings support the hypothesis that ApoE4 role is more visible in the early stages of AD rather than in more advanced stages. (2) All diagnoses in our sample were made clinically at follow-up, but not confirmed histopathologically nor confirmed by biomarker results. This may cause some diagnostic inaccuracy, and thus disease-specific mechanisms of ApoE4 remain speculative. Still, the lack of a significant interaction of ApoE4 carriership with diagnosis on CSF biomarker levels does not suggest any AD-specific mechanisms of ApoE4 on biomarkers. (3) The DCN sample is not a population-based cohort, rather it is more representative of real clinical setting of specialized memory clinic patients, what may be a strength of the study when considering clinical diagnostic practice. (4) Although AD core CSF biomarkers of amyloid, phospho-tau and total tau are currently well validated measures of AD pathology, this study should be replicated using modern neuroimaging techniques such as in vivo amyloid and Tau PET 5) Lastly, due to practical reasons, (i.e., clinical progression to dementia which limits the possibility for memory clinic visits), we applied a relatively short follow-up period with a mean of 2.5 years, implying that a certain proportion of patients who were classified as stable are likely to progress to dementia later on. We strongly encourage that the confirmation of these finding in larger sample, with longer follow-up time, or with higher conversion rate to AD, should be carried out.

In summary, we confirmed the powerful ApoE4 dosage-dependent effect on CSF Aβ42 levels reported previously, add data on a positive association between ApoE4 allele number and both, CSF t-tau and p-tau levels, with an effect independent of  CSF Aβ42 in amyloid-negative subjects, and show that ApoE genotype affects speed of clinical progression in AD. The data also may indicate that in clinical AD at the stage of MCI, the impact of ApoE4 is modified by other mechanisms of neurodegeneration, with implications for diagnostic utility. Thus, our results contribute to an emerging framework in which ApoE4 involves general mechanisms associated with both CSF amyloid-β burden and tau aggregation at specific time points in AD pathogenesis.

## Data Availability

Anonymized data will be made available to the scientific community upon request.
